# Quack Advertisements and the Religious Press

**Published:** 1881-06

**Authors:** 


Quack Advertisements, and the Religious Press.—It is gratifying to learn from some of our contemporaries, that a few of the religious newspapers have ceased to pander to the wishes and interests of nostrum venders of every sort, and having purged their advertising columns of the vile impostures, proclaiming the miraculous cures of cases, that many times never occurred, have set their faces firmly against the admission of such stuff into their papers in the future. We would be happy if we could say as much for the religious press of some other communities we wot of; but a proper regard for truth compels us to say we cannot. Shall we do evil that good may come? might with great propriety be asked the editor, or owner of a religious newspaper, who publishes on *any* account, and especially for his *pecuniary* advantage, the advertisement of a quack nostrum, of no value, and often productive of actual harm to the person who uses it? We are aware that it requires money to run a religious publication as well as a medical, or any other one; but the possessor of pure and undefiled religion is not likely to lend his aid and influence for the perpetration of wrong and harm to his fellow men, merely that he may enrich his coffers or put money in his pocket. Such a course is not only in contravention of the animus, but also of the very letter of the law of the meek and lowly one of Nazareth. We hope, therefore, our brethren of the medical press will continue to cry down, and spare not, those clergymen who publish quack advertisements for money in their newspapers, or give the sanction of their names to advance the interests of those engaged in any way in the nefarious business of swindling unsuspecting and credulous sufferers, from real and imaginary ills, with worthless or vile compounds of any sort? Wanting, as the medical press may be, in some respects, it is gratifying to be able to record the fact that no reputable medical journal can be induced to countenance or advertise secret nostrums, and quack preparations, knowingly, for money. Will our religious confreres of the "fourth estate" take due notice of this fact, and henceforth govern themselves accordingly?

